# Quantitative Proteomic and Transcriptomic Study on Autotetraploid Paulownia and Its Diploid Parent Reveal Key Metabolic Processes Associated with Paulownia Autotetraploidization

**DOI:** 10.3389/fpls.2016.00892

**Published:** 2016-06-24

**Authors:** Yanpeng Dong, Minjie Deng, Zhenli Zhao, Guoqiang Fan

**Affiliations:** ^1^Department of Forestry, College of Forestry, Henan Agricultural UniversityZhengzhou, China; ^2^Institute of Paulownia, Henan Agricultural UniversityZhengzhou, China

**Keywords:** proteome, transcriptome, autotetraploid, Paulownia, iTRAQ

## Abstract

Polyploidy plays a very important role in speciation and plant evolution by way of genomic merging and doubling. In the process of polyploidy, rapid genomic, and transcriptomic changes have been observed and researched. However, proteomic divergence caused by the effects of polyploidization is still poorly understood. In the present study, we used iTRAQ coupled with mass spectrometry to quantitatively analyze proteomic changes in the leaves of autotetraploid Paulownia and its diploid parent. A total of 2963 proteins were identified and quantified. Among them, 463 differentially abundant proteins were detected between autotetraploid Paulownia and its diploid parent, and 198 proteins were found to be non-additively abundant in autotetraploid Paulownia, suggesting the presence of non-additive protein regulation during genomic merger and doubling. We also detected 1808 protein-encoding genes in previously published RNA sequencing data. We found that 59 of the genes that showed remarkable changes at mRNA level encoded proteins with consistant changes in their abundance levels, while a further 48 genes that showed noteworthy changes in their expression levels encoded proteins with opposite changes in their abundance levels. Proteins involved in posttranslational modification, protein turnover, and response to stimulus, were significantly enriched among the non-additive proteins, which may provide some of the driving power for variation and adaptation in autopolyploids. Quantitative real-time PCR analysis verified the expression patterns of related protein-coding genes. In addition, we found that the percentage of differentially abundant proteins that matched previously reported differentially expressed genes was relatively low.

## Introduction

Polyploidy is a widespread and prominent process that plays an important role in the evolution of all angiosperm plants through genomic merging and doubling. There are three main types of polyploids: autopolyploids, allopolyploids, and segmental allopolyploids (Stebbins, 1947). Autopolyploids such as *Solanum tuberosum, Saccharum officinarum*, and *Medicago sativa*, combine more than two sets of identical genomes in their nucleus (Chen, 2010). They often thrive more vigorously and can survive in severe environments because, through genome duplication (Soltis and Soltis, 2009), they have traits that are superior to their parents. Many hypotheses have been proposed to explain the function of a doubled genome. One such hypothesis is that genome duplication endows distinct superiorities that enable polyploids to survive and thrive in circumstances that are too severe for their parents (Otto, 2007; Weiss-Schneeweiss et al., 2013). Despite the widespread occurrence and survival superiorities of polyploids, and some progress in this area, there is still not enough information to explain the cause and direct effect of the assigned biological functions of the increased content of the doubled genome.

High-throughput genomic and transcriptomic sequencing techniques have contributed greatly to gaining a comprehensive perspective on the genomic information of autopolyploids. However, because changes at the transcriptional level do not always correlate with changes at the translational level, it is also necessary to investigate the differences between autotetraploids and their corresponding progenitors at the proteome level. The correlation between the expression of RNA transcripts and protein abundance is usually not direct because processes such as posttranscriptional regulation and posttranslational modifications can make it difficult to predict patterns of protein abundance. Thus, applying proteomic approaches to investigate autopolyploids will greatly increase the understanding of their evolution and adaption. Until recently, proteomic changes in autopolyploids and their diploid parents have been rarely reported, and only a few studies on *Arabidopsis* (Ng et al., 2012) and *Manihot esculenta* Crantz (An et al., 2014) were found. In most of these researches, the method of two-dimensional electrophoresis (2-DE) combined with mass spectrometry was used. In general, there are three kinds of methods involving in proteome: 2-DE, label, and shotgun. They are complementary to each other. Coupled with mass spectrometry analysis, iTRAQ has proven to be a powerful method to investigate proteomic changes because many proteins can be identified simultaneously and proteomic changes can be measured with high sensitivity in related species (Ross et al., 2004; O'Brien et al., 2010). Therefore, an iTRAQ-based proteomic analysis in autopolyploids should provide valuable information and new insights into the effects of autopolyploidization at the translational level (Koh et al., 2012).

Paulownia is an economically important genus of trees that are native to China. Paulownia “*Yuza 1*” is an improved species that was bred from *P. tomentosa* × *P. fortunei*. The autopolyploid “*Yuza 1*” does not exist in nature; however, it has valuable traits, including drought and witches' broom diseases resistance, that were acquired as a result of its autopolyploidy (Liu et al., 2013; Li et al., 2014; Xu et al., 2014). In this study, iTRAQ technology combined with liquid chromatography coupled with tandem mass spectrometry (LC-MS/MS) was employed to investigate the effects of polyploidization on the “*Yuza 1*” proteome. Our results provide a survey of protein changes as a result of genome duplication and provide a better understanding of the mechanisms of autoploidization in Paulownia. We also cataloged differences in the abundance of proteins and expression levels of mRNAs using previously reported RNA sequencing data (Li et al., 2014). Finally, potential target genes identified in this study could be engineered for acclimation in plants, especially stress.

## Materials and methods

### Plant material

All biological materials used in this study were obtained from the Institute of Paulownia, Henan Agricultural University, Zhengzhou, Henan Province, China.

Uniformly grown tissue cultured seedlings of the “*Yuza 1*” diploid (Y2, “*Yuza 1*” diploid) and autotetraploid (Y4, “*Yuza 1*” autotetraploid) were cultured in 100-mL triangular flasks on 1/2 MS medium for 30 days. Healthy leaves were harvested, and equal numbers of leaves from each of the three plants in each of the replicate groups [Y2, Y2-2 (replicates for “*Yuza 1*” diploid)]; [Y4,Y4-2 (replicates for “*Yuza 1*” autotetraploid)] were pooled to form the two samples. Terminal buds of 1.5 cm in length of the plantlets in the four treatment groups were sheared, immediately frozen in liquid nitrogen, and stored at −80°C.

### Protein extraction, digestion, and iTRAQ labeling

The Y2 and Y4 samples were ground into powder in liquid nitrogen, extracted with lysis buffer containing 1 mM PMSF and 2 mM EDTA. After that, protein was extracted according to the method of Tang et al. (2016). Total protein was digested and iTRAQ-labeled according to the method of Meng et al. (2014). The samples were labeled with iTRAQ tags as follows (sample, tag): Y2, 113; Y4, 116; Y2-2, 117; Y4-2, 118. Four other samples were labeled with the same tags as repeats. The peptides were labeled with the isobaric tags and incubated at room temperature for 2 h. The labeled peptide mixtures were then pooled for strong cation exchange and dried by vacuum centrifugation.

### Strong cation exchange

Strong cation exchange chromatography was performed with a LC-20AB HPLC pump system. The iTRAQ-labeled peptide mixtures were reconstituted with 4 mL buffer A and loaded onto a 4.6 × 250 mm Ultremex SCX column containing 5-μm particles. The peptides were eluted at a flow rate of 1 mL min^−1^ with a gradient of buffer A for 10 min, 5–60% buffer B for 27 min, and 60–100% buffer B for 1 min. The system was then maintained at 100% buffer B for 1 min before equilibrating with buffer A for 10 min prior to the next injection. Elution was monitored by measuring the absorbance at 214 nm, and fractions were collected every 1 min. The eluted peptides were pooled into 20 fractions, desalted with a Strata X C18 column and vacuum-dried.

### LC-MS/MS analysis

Mass spectroscopy analysis was performed using an AB SCIEX TripleTOF™ 5600 mass spectrometer, coupled with an online micro-flow HPLC system as described in Section Strong Cation Exchange. The peptides were separated using the method of Qiao et al. (2012).

### iTRAQ protein identification and quantification

The acquired raw data files were converted into Mascot generic format files according the method of Lin et al. (2013). Protein identification was performed using the Mascot search engine against a Paulownia transcriptome database that contained 82,934 sequences. The sequencing data have been submitted to the Short Reads Archive under accession number SRP034738 (Li et al., 2014). Protein identification and quantification was performed according the method of Guo et al. (2014).

To reduce the probability of false peptide identification, only peptides with significance scores in the 99% confidence interval greater than “identity” in a Mascot probability analysis were counted as identified. Each confident protein identification involved at least one unique peptide.

For protein quantization, each protein was required to contain at least two unique peptides. The quantitative protein ratios were weighted and normalized by the median ratio in Mascot. Only ratios with *p* < 0.05 and fold changes >1.2 were considered significant.

### Bioinformatics analysis

Functional analysis of the identified proteins was conducted using Gene Ontology annotations and the proteins were categorized according to their biological processes, molecular functions and cellular localizations. The proteins were further analyzed using the Clusters of Orthologous Groups of proteins database and the Kyoto Encyclopedia of Genes and Genomes database (Fan et al., 2014). Principal Component Analysis (PCA) of the samples were done using the software of SIMCA-P(Wu et al., 2010).

### RNA preparation and quantitative RT-PCR

The RNA samples from Y2, Y4, Y2-2, and Y4-2 were extracted with Trizol (Sangon, Shanghai, China). The RNA was then precipitated with isopropanol. Purified and concentrated RNA was denatured and first-strand cDNAs for all the samples were synthesized using a PrimeScript RT reagent Kit (Takara, Dalian, China). Primers were designed using Beacon Designer version 7.7 (Premier Biosoft International, Ltd., Palo Alto, CA, USA; Table 1). The genes encoding 10 differentially abundant proteins (DAPs) were selected for qRT-PCR analysis. The qRT-PCR reactions were run in So Fast EvaGreen Supermix starting with 1 μL of the cDNA template in a standard 20-μL reaction. The cDNAs were then amplified in a Bio-Rad CFX96TM Real-Time System with SYBR Premix Ex Taq TM II. The PCR cycles were as follows: 95°C for 1 min, followed by 40 cycles of 95°C for 10 s, and 55°C for 15 s. Relative expression levels of the genes were calculated using the 2^−ΔΔCt^ method and normalized with 18S rRNA from “*Yuza 1*.”

**Table 1:** **Primers of quantitative RT-PCR analysis of candidate DAP genes**.

**Unigene-ID**	**Potential gene function**	**Size (bp)**	Up/down^*^	**Primer sequence**
CL2443.Contig2_All	Translation initiation factor 1A	2332	Up	ATTCGTGAGCATTCGCCATTC
				AGCATTGTGAGCCAGGAACC
CL1351.Contig2_All	GTP-binding protein SAR1A	372	Up	GAAGGCAATAACAGGAGGAG
				GTTGATGGCAGCAGTTCC
Unigene18898_All	Pectin methyl-esterase	474	Up	TGCTGCTCCTAATCTCTAAAC
				CATCCTTGTGATACTTTGTGAC
CL4141.Contig1_All	Photosystem I subunit III	1528	Up	CTCTCCTTACTGCCATCTC
				CGTATCTTGTGTTTATGTTAGC
Unigene20285_All	Ubiquinol-cytochrome C reductase	422	Up	AGGACTCTGAATAGAACTTATGG
				TTGACTCCACTACTAACATAGC
CL2464.Contig1_All	Histone H4	2099	Up	ATCAATCGGCGGAATAGGC
				AGATGCGGTCACTCACAAC
Unigene4043_All	Lipid transfer protein 2	575	Up	ATGGACACGAGAATAAGG
				TGGTAATTGTAGTTGTTAGG
CL4400.Contig1_All	Photosystem I reaction center subunit XI	1239	Up	TGCGATACTGACACAACTAAG
				AACTCAACATTACCACCTTCC
CL9028.Contig1_All	Nitrate excretion transporter 1	1067	Up	GACTTATGCTCTGGTTGTTG
				AGATTCGGTGAGGCTAATAC
CL5761.Contig1_All	Vacuolar ATP synthase subunit G1	778	Up	GTGTTCCAAGTCATTAGCC
				TGTCAAAGAAAGAAGAAATACG
Unigene4328_All	Monocopper oxidase-like protein SKS1	1138	Up	TTACATCCACAGTTTCACAAATC
				GCCTTCCACTTCACATTCC
CL4582.Contig1_All	Monocopper oxidase-like protein SKU5 OS	4768	Up	TGCCAACGCCTCCACAAC
				CCTCCCAGTCTACTACGAAACC
CL3509.Contig2_All	Fructose-2,6-bisphosphatase	2087	down	TGGAGGCGGTGGTTGTTG
				AGCAAGGAAGCATCATTCAAGG
CL8969.Contig1_All	Pathogenesis-related protein 10.4	955	down	ACGACGACATAATGATGGAAAC
				CGATGCCGAGGACGATTC
CL392.Contig1_All	Brassinosteroid-regulated protein BRU1	1588	down	CTCTGACAATGTTCCTTATGATGC
				CTGTAGGCTCCGAATGTAATGG
CL3302.Contig1_All	Acetylajmalan acetylesterase	3003	down	GCTGGTGCCTGGTGATATG
				GATGGAGTGACAGTGGTTGG
CL2061.Contig4_All	Translation initiation factor IF-2	1191	down	AATAGCATAGACCAGACAGC
				AAGGAATCAAAGAAGAAGAAGG

* *Up or down regulation indicated the expression in the Y4 vs. Y2 comparison*.

## Results

### Proteomics characterization

**Figure 1:**
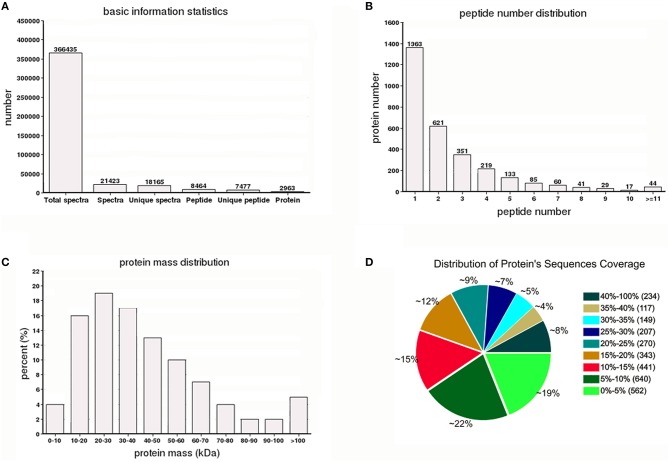
**Summary of the iTRAQ-based proteome. (A)** Spectra, peptides, and proteins that were identified from iTRAQ proteomics by searching against the “*Yuza 1*” transcriptome database. **(B)** Number of peptides that match proteins using MASCOT. **(C)** Distribution of the proteins that were identified among different molecular weights. **(D)** Coverage of the proteins by the identified peptides.

**Figure 2:**
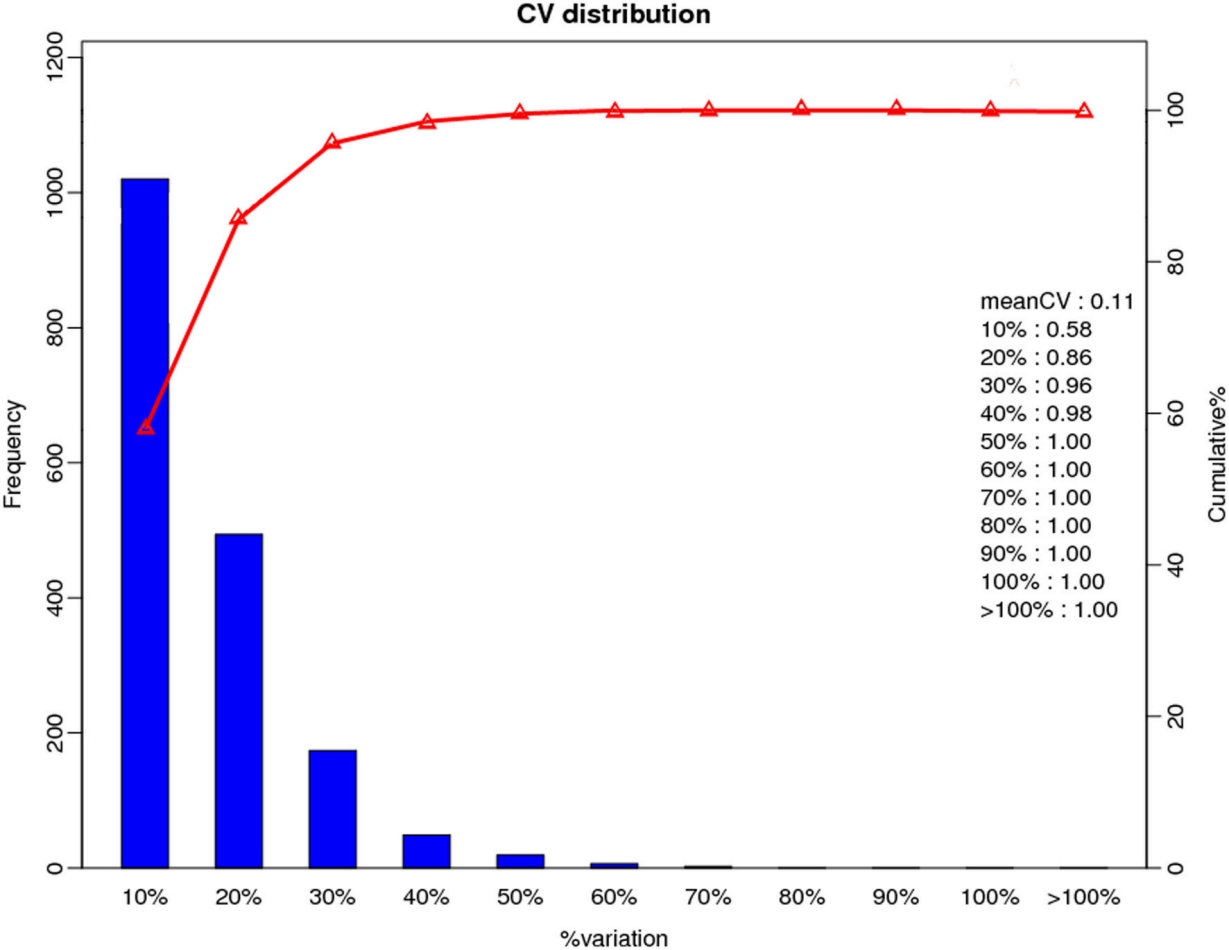
**Repeatability of the expression of duplicate samples**. X-axis represents the difference of the quantitative ratios between the first and the second biological repeats of the two samples. The right y-axis represents the cumulative percentage between the proteins of a certain range and the quantitative proteins, while the left y-axis represents the number of total protein in a certain range.

A total of 366,435 spectra were generated from the iTRAQ-based quantitative proteomics analysis of the proteins extracted from Y2, Y2-2, Y4, and Y4-2 seedling leaves. The analysis using Mascot software identified 21,423 spectra that matched known spectra. Among them, 18,165 unique spectra were matched to 7477 unique peptides and 2963 proteins (Figure 1A), ~54% of which consisted of at least two of the peptides (Figure 1B). The vast majority of these proteins were larger than 10 kDa, although their molecular weights covered a wide range (Figure 1C). Most of the identified proteins had good peptide coverage; 59% had more than 10% sequence coverage, and 33% had 20% sequence coverage (Figure 1D). Further, to avoid identification omissions, we also confined the peptide matching error of the database search strategy to less than 2 ppm. The reproducibility of the proteomic analysis is shown in Figure 2. These results indicate that the proteomics analyses were reliable. The results of PCA of the samples were shown in Figure S1. The mass spectrometry proteomics data have been deposited to the ProteomeXchange Consortium via the PRIDE (Vizcaíno et al., 2016) partner repository with the dataset identifier PXD004237. Data are available via ProteomeXchange with identifier PXD004237 (Vizcaino et al., 2014). Reviewer account details were as follows: username was reviewer36112@ebi.ac.uk and password was TH1qVbgd.

**Figure 3:**
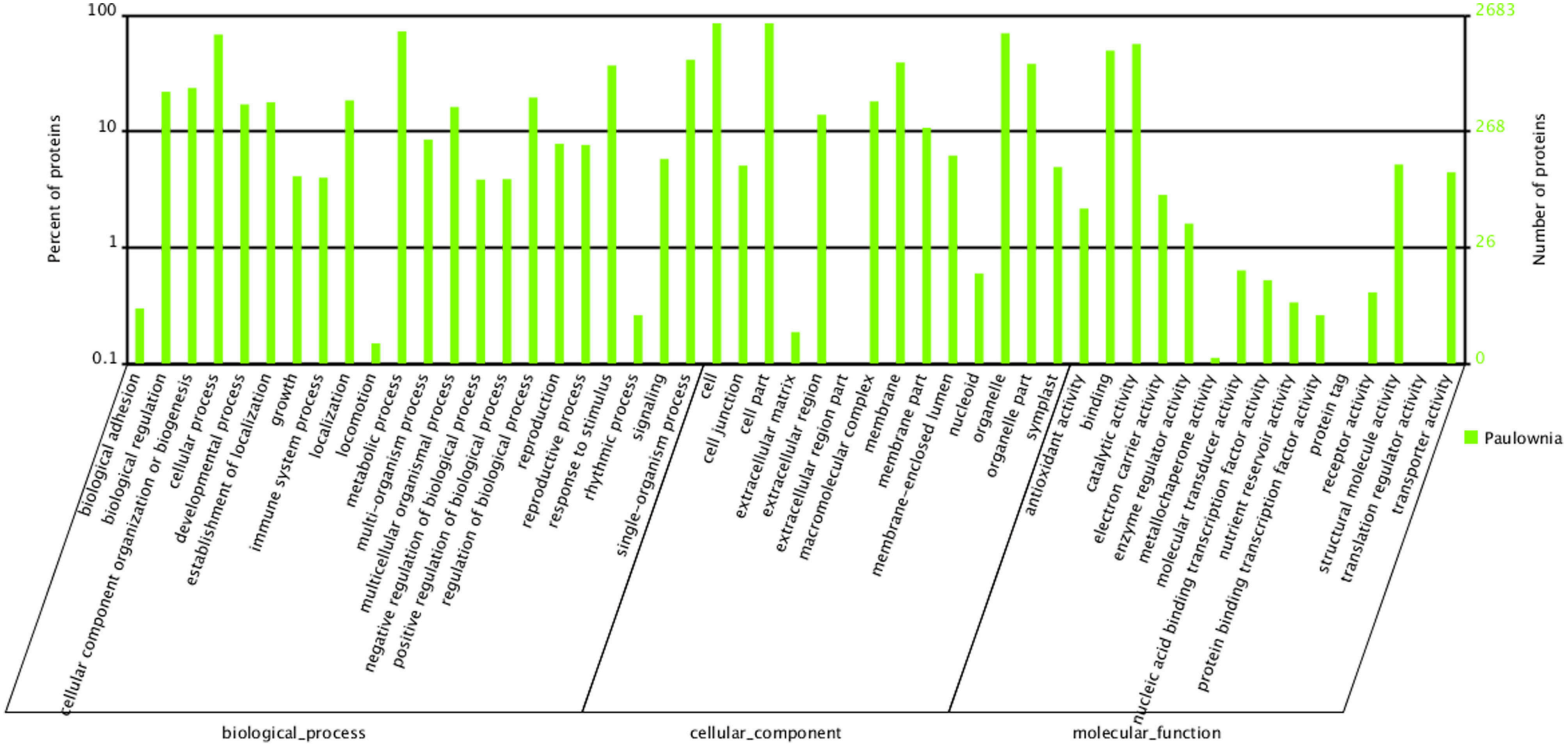
**Gene Ontology classification of distinct proteins that were detected in “***Yuza 1***” leaves 2683 proteins (90.55% of total) were categorized into 51 function groups**.

To carry out a functional analysis, we assigned GO terms to all the quantitated proteins. Under biological process, metabolic process (1950) and cellular process (1830) were the most represented groups; under cellular component, cell (2290) and cell part (2290) were the two largest groups; and under molecular function, catalytic activity (1518) and binding (1331) were most represented (Figure 3). These results indicate that the identified proteins are involved in almost every aspect of “*Yuza 1*” metabolism.

**Figure 4:**
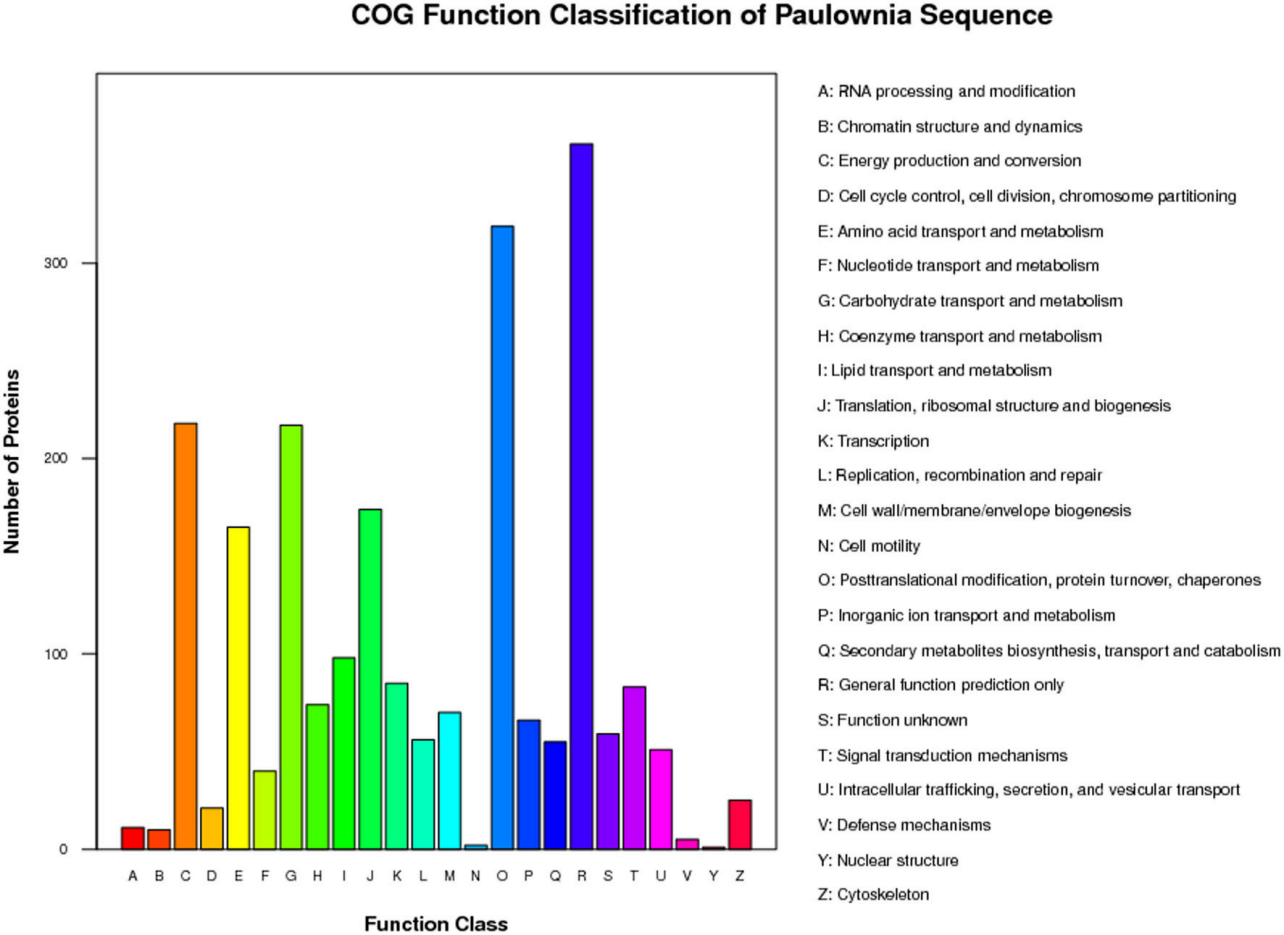
**COG function classification of distinct proteins that were detected in “***Yuza 1***” leaves protein or domains were annotated and divided into 23 specific categories**.

To further understand the functions of the 2963 proteins, they were assigned to 23 categories using the COG database. The most highly represented functional categories were “general function prediction only” and “posttranslational modification, protein turnover, chaperones,” which represented ~16 and 14% of the identified proteins, respectively (Figure 4).

Next, the annotated proteins were mapped onto 121 KEGG pathways (Table S1). Among them, “metabolic pathways” (758) was significantly more highly represented than the other pathways, and was followed by “biosynthesis of secondary metabolites” (455) and “ribosome” (116). Based on the KEGG analysis, we concluded that most of the mapped proteins may affect cellular component biogenesis, posttranslational modification, protein turnover, and response to stimulus.

### Overview and analysis of differentially abundant proteins related to autotetraploidy

DAPs were defined as proteins that showed a greater than 1.2-fold change in relative abundance and a *p* < 0.05. A total of 463 DAPs were identified in the Y4 vs. Y2 comparison; 265 were more abundant and 198 were less abundant (Table S2). Among the top 10 more abundant DAPs, only five could be aligned to proteins with known functions in GenBank. These proteins were annotated as lipid transfer protein 2 (*Salvia miltiorrhiza*, ABP01769.1gi|144601657), 40S ribosomal protein S20-2 isoform 1 (*Vitis vinifera*, XP_002265347.1), phenylcoumaran benzylic ether reductase homolog Fi1 (*Forsythia* × *intermedia*, AAF64174.1), glucose-6-phosphate dehydrogenase (*Capsicum annuum*, AAT75322.1), and chlorophyllase (*Sesbania rostrata*, BAG55223.1). Among the top 10 less abundant proteins, eight could be aligned to proteins with known functions in GenBank. These proteins were annotated as acetylajmalan acetylesterase (*Striga asiatica*, ABD98038.1), glyoxalase I (*Avicennia marina*, AAK06838.1), acetylajmalan acetylesterase (*Striga asiatica*, ABD98038.1), brassinosteroid-regulated protein BRU1 (*V. vinifera*, XP_002270375.1), methyltransferase DDB_G0268948 (*Solanum lycopersicum*, XP_004237334.1), protein VITISV_028080 (*V. vinifera*, CAN70727.1), miraculin-like protein (*V. vinifera*, XP_002266302.2), and PRUPE_ppa005409mg (*Prunus persica*, EMJ12790.1). Interestingly, proteins playing important roles in paulownia stress tolerance were found in the comparison of Y4 vs. Y2. These proteins include XP_002266352.1, XP_002329905.1, XP_004234931.1, XP_002513962.1, AFA35119.1, XP_004229610.1, XP_004234945.1, XP_002512963.1, XP_002263538.1, XP_004146530.1, CAA54303.1, XP_004236869.1, XP_004246134.1, ABJ74186.1, AEO19903.1, XP_002276841.1, AFU95415.1, ABB16972.1, AFH08831.1, NP_178050.1, XP_002333019.1, AAK06838.1, XP_004234985.1, P32980.1, AEA86324.1, CBI20688.3, ABK32073.1, ABK55669.1, XP_002320236.1, AFC01205.1, CAA06961.1, EMJ16250.1, ABF97414.1, AEO19903.1, AFA35119.1, CBI20065.3, O49996.1, CAJ00339.1, ADK70385.1, NP_001117239.1, ABK32883.1, CAJ19270.1, CAJ43709.1, XP_003626827.1, BAF62340.1, CAA82994.1, AEO45784.1, BAA89214.1, CAN74175.1, CAM84363.1, ABK94455.1, CBI24182.3, XP_002519217.1, XP_004251036.1, CAC43323.1, BAD02268.1, CAH17549.1, XP_002298998.1, ABS70719.1, CAH17986.1, XP_002513962.1, AFK42125.1, AFP49334.1, EMJ28736.1, XP_002276841.1, XP_002315944.1, CAC43318.1, P84561.1, CBI34207.3, AEG78307.1, CAH58634.1, AFM95218.1, AFJ42571.1, BAD10939.1, CBI21031.3, XP_002305564.1, XP_002313736.1, XP_004237334.1, YP_636329.1, AFJ42575.1, NP_001234431.1.

### Comparison of the abundance profiles of DAPs between the Y2 and Y4 samples

**Figure 5:**
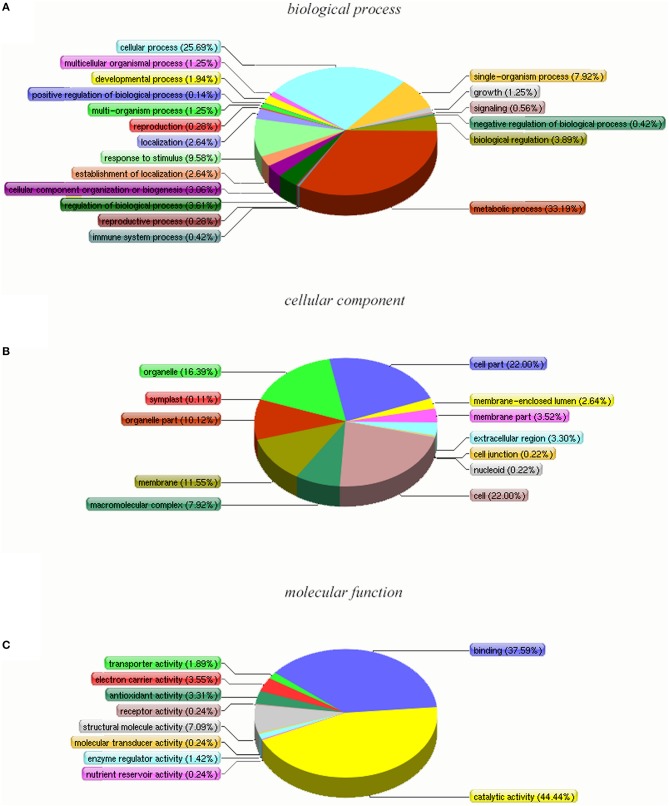
**Gene Ontology (GO) classification of DAPs that were detected in “***Yuza 1”*** leaves 321 proteins (69.33% of total) were categorized into 41 function groups**. **(A)** Biological process of GO; **(B)** cellular component of GO; **(C)** molecular function of GO.

Based on the Y4 vs. Y2 comparison, DAPs were retrieved and analyzed using the GO, COG, and KEGG databases. The DAPs were assigned to 51 functional groups under the three main GO categories (Figure 5 and Table S3). Under biological process, metabolic process and cellular process were the most represented groups; under cellular component, cell and cell part were the two largest groups; and under molecular function, catalytic activity and binding were the most represented. Interestingly, the GO classifications for the DAPs were similar to the classifications for all the identified proteins. The most enriched GO terms under biological process, cellular component, and molecular function were the same for all the proteins and for the DAPs subset (Table 2). We suggest that these enriched GO terms may be related to autotetraploidy.

**Table 2:** **Statistical analysis of the biological process, cell component and molecular function of the differentially abudant proteins (DAPs) in the Y4 vs. Y2 comparison**.

**Ontology**	**Class**	**DAP-Number**
Biological process	Biological regulation	28
	Cellular component organization or biogenesis	22
	Cellular process	185
	Developmental process	14
	Establishment of localization	19
	Growth	9
	Immune system process	3
	Localization	19
	Metabolic process	239
	Multi-organism process	9
	Multicellular organismal process	9
	Negative regulation of biological process	3
	Positive regulation of biological process	1
	Regulation of biological process	26
	Reproduction	2
	Reproductive process	2
	Response to stimulus	69
	Signaling	4
	Single-organism process	57
Cellular component	Cell	200
	Cell junction	2
	Cell part	200
	Extracellular region	30
	Macromolecular complex	72
	Membrane	105
	Membrane part	32
	Membrane-enclosed lumen	24
	Nucleoid	2
	Organelle	149
	Organelle part	92
	Symplast	1
Molecular function	Antioxidant activity	14
	Binding	159
	Catalytic activity	188
	Electron carrier activity	15
	Enzyme regulator activity	6
	Molecular transducer activity	1
	Nutrient reservoir activity	1
	Receptor activity	1
	Structural molecule activity	30
	Transporter activity	8

To predict and classify the possible functions of the DAPs, we assigned them to COG categories. Based on their sequence homology with known proteins in GenBank, 355 DAPs, which comprised 11.98% of all the proteins, were divided into 20 categories (Table 3). The “posttranslational modification, protein turnover, chaperones” category contained 48 proteins and was the largest, followed by “energy production and conversion” (47), “translation, ribosomal structure and biogenesis” (46), “general function prediction only” (41), “carbohydrate transport and metabolism” (38), “amino acid transport and metabolism” (27), and “coenzyme transport and metabolism” (17). The smallest group was “intracellular trafficking, secretion, and vesicular transport” with only one protein (Figure S2).

**Table 3:** **Statistics of the functional categories of the DAPs that were detected in “***Yuza 1***” leaves**.

**Code**	**Functional-categories**	**DAP-number**
B	Chromatin structure and dynamics	2
C	Energy production and conversion	47
D	Cell cycle control, cell division, chromosome partitioning	2
E	Amino acid transport and metabolism	27
F	Nucleotide transport and metabolism	6
G	Carbohydrate transport and metabolism	38
H	Coenzyme transport and metabolism	17
I	Lipid transport and metabolism	13
J	Translation, ribosomal structure and biogenesis	46
K	Transcription	9
L	Replication, recombination and repair	4
M	Cell wall/membrane/envelope biogenesis	13
O	Posttranslational modification, protein turnover, chaperones	48
P	Inorganic ion transport and metabolism	17
Q	Secondary metabolites biosynthesis, transport and catabolism	4
R	General function prediction only	41
S	Function unknown	8
T	Signal transduction mechanisms	9
U	Intracellular trafficking, secretion, and vesicular transport	1
Z	Cytoskeleton	3

The DAPs were mapped to 77 KEGG metabolic pathways (Table S4): “metabolic pathways,” “biosynthesis of secondary metabolites,” “ribosome,” and “photosynthesis” were highly enriched, followed by “glyoxylate and dicarboxylate metabolism,” “amino sugar and nucleotide sugar metabolism,” “pyruvate metabolism,” “carbon fixation in photosynthetic organisms,” “other glycan degradation,” “glycolysis/gluconeogenesis,” “starch and sucrose metabolism,” and “protein processing in endoplasmic reticulum.”

### Correlation between proteins and transcripts

To compare changes in protein abundance with transcript levels, we compared the more and less abundant DAPs identified by iTRAQ with the up- and down-regulated differentially expressed genes identified from a previous transcriptome analysis. Differentially expressed unigenes were screened based on an absolute fold change value of log2 ratio >1 with *p* < 0.001 and FDR < 0.001. A total of 1808 corresponding protein-encoding genes were detected by RNA sequencing. We found that 59 of the genes that showed noteworthy changes in their expression levels encoded proteins with corresponding changes in their abundance levels; 37 of these genes were up-regulated and 22 were down-regulated, while the corresponding proteins were more and less abundant, respectively (Table S5). A further 48 genes that showed noteworthy changes in their expression levels encoded proteins with opposite changes in their abundance levels (Table S5). We found 252 genes that showed noteworthy changes in transcript levels, while the corresponding proteins showed no changes in their abundances (Table S6). Conversely, 356 proteins showed noteworthy changes in their abundance levels, while the corresponding encoding genes showed no changes in their expression levels (Table S7).

### Confirmation of differentially expressed proteins by qRT-PCR

**Figure 6:**
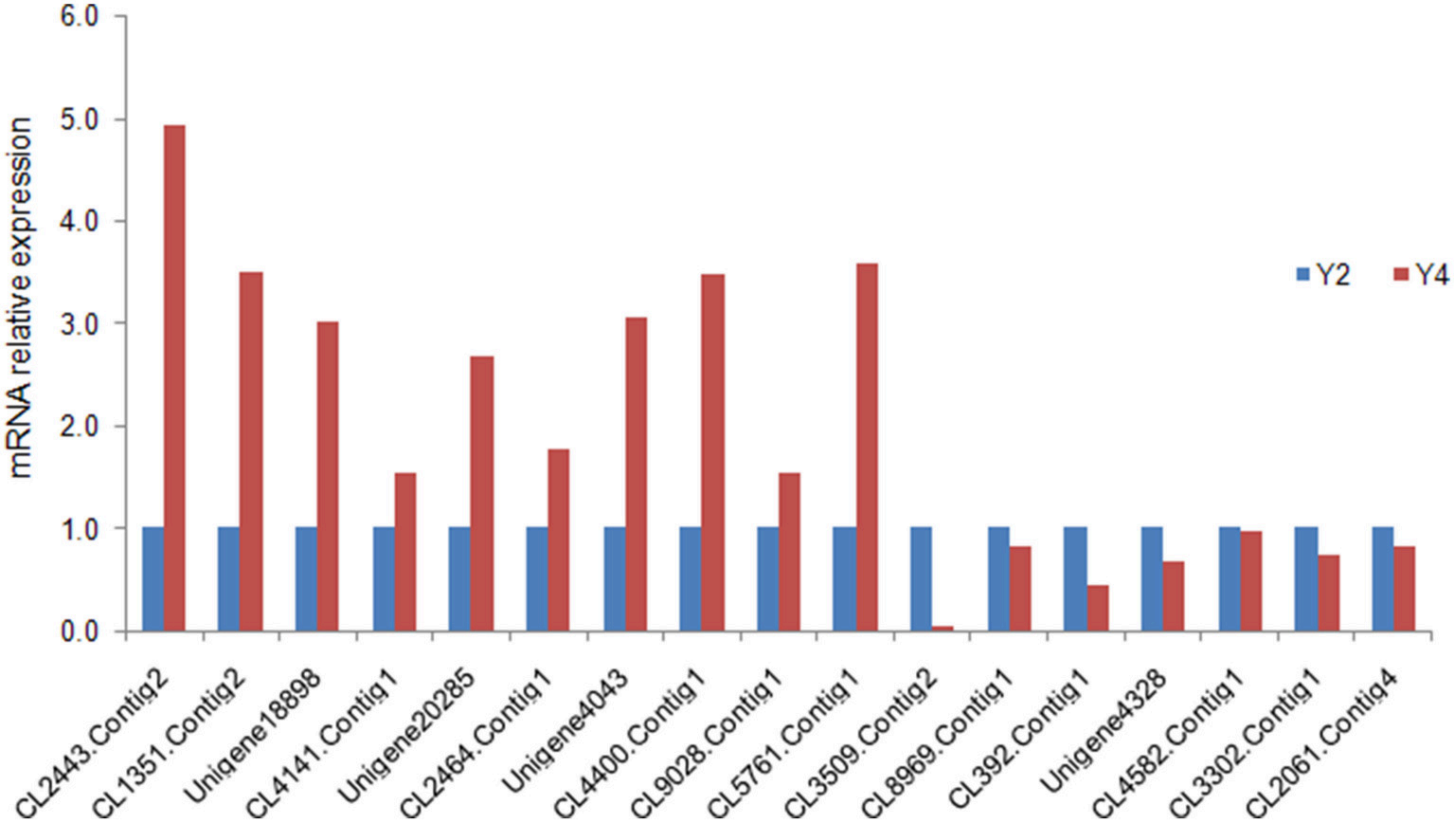
**Quantitative RT-PCR analysis of “***Yuza 1”*** candidate proteins**. The 18S rRNA of Paulownia was chosen as an internal reference gene for normalization. CL2443.Contig2_All: translation initiation factor 1A; CL1351.Contig2_All: GTP-binding protein SAR1A; Unigene18898_All: pectin methyl-esterase; CL4141.Contig1_All: photosystem I subunit III; Unigene20285_All: ubiquinol-cytochrome C reductase; CL2464.Contig1_All: histone H4; Unigene4043_All: lipid transfer protein 2; CL4400.Contig1_All: photosystem I reaction center subunit XI; CL9028.Contig1_All: nitrate excretion transporter 1; CL5761.Contig1_All: vacuolar ATP synthase subunit G1; CL3509.Contig2_All: fructose-2,6-bisphosphatase; CL8969.Contig1_All: pathogenesis-related protein 10.4; CL392.Contig1_All: brassinosteroid-regulated protein BRU1; Unigene4328_All: monocopper oxidase-like protein SKS1; CL4582.Contig1_All: monocopper oxidase-like protein SKU5 OS; CL3302.Contig1_All: acetylajmalan acetylesterase; CL2061.Contig4_All: translation initiation factor IF-2.

To confirm the differential expression of proteins identified by iTRAQ analysis, qRT-PCR was performed to detect the transcript expression levels of the corresponding genes. In Y4 vs. Y2, the qRT-PCR results shown that 15 DAPs were consistent with the iTRAQ LC-MS/MS analysis results, 2 DAPs displayed opposite protein and mRNA expression patterns (Figure 6), 1 transcript of the DAP failed to be amplified in the qRT-PCR experiment. This discrepancy may be attributed to posttranscriptional and posttranslational regulatory processes.

## Discussion

### Insights into the proteomic and transcriptomic analyses of autopolyploids

An important aspect of polypoid genomics research is the observation of genome expansion and contraction. The *C*-value, which is a measure of the amount of DNA in the unreplicated gametic nucleus, is often used to estimate the ploidy level in plants (Udall et al., 2006). It is assumed that polyploids have large C-values that increase in direct proportion with ploidy (Poggio et al., 2014). However, all three possible fates of genome size evolution after ploidization, namely expansion, contraction, and no change, have been reported. Genome expansion has been reported in only a few studies. In a recent study of five *Nicotiana* polyploids, the genomes of the polyploids were found to have increased in size (Leitch and Leitch, 2008). Conversely, a large-scale analysis of 3008 angiosperms that combined available genome size data, suggested that genome contraction was a widespread biological response after polyploid formation (Leitch and Bennett, 2004; Ammiraju et al., 2010). It is also been reported that in some polyploid series, there is no change in genome size after polyploidization, and such phenomenon especially takes plase in newly-formed ones (Yang et al., 2011). In our work, the genome size of the autotetraploid Paulownia was found to be less than twice the diploid genome size, suggesting genome contraction had occurred in the process of autopolyploidization. We predicted that the contraction may be due to an insertion/deletion bias that could result in frequent and larger deletions and result in a reduction in genome size because of an increase in illegitimate recombinations (Grover et al., 2007).

Our proteomic and transcriptomic data gave quite different abundance/expression results. This may be because gene expression differences were not translated into protein abundance differences because of posttranscriptional regulation and/or posttranslational modification, or because differences between the autotetraploid and its diploid parent were underestimated at the proteomic level because the iTRAQ-based analysis determines the number of DAPs based on an estimate of the fold change of different samples. It has been reported that iTRAQ-based methods may have problems with accuracy because they may systematically underestimate ratios, especially when the ratio changes are large (Bantscheff et al., 2008; Ow et al., 2009; Karp et al., 2010). In this work, as in most iTRAQ studies, fold changes ≥1.2 or ≤ 0.8 were employed because, in iTRAQ quantitation, small changes may reflect larger differences.

### Regulation of protein expression in “*Yuza 1”* autotetraploid can increase the possibility of adaptation to various stresses

Polyploids have been reported to acquire enhanced stress responses compared with the corresponding diploids (Saleh et al., 2008; Chandra and Dubey, 2010; Manzaneda et al., 2012). Casneuf et al. found through a genome-wide analysis in Arabidopsis, that stress-related genes were more likely to be retained during polyploidization compared with other genes (Casneuf et al., 2006). In a previous study, we also found that genes involved in stress responses, including drought, salt, and phytoplasma infection, were significantly up-regulated in Paulownia autotetraploids compared with their diploid parents (Liu et al., 2013; Dong et al., 2014a,b,c; Fan et al., 2014; Xu et al., 2014). Moreover, the regulation of microRNA expression in Paulownia autotetraploids also increased the probability of adaptation to climate because some down-regulated microRNAs were found to target genes that were related to response to stress in autotetraploids compared with their parents (Fan et al., 2015; Niu et al., 2016). In this study, we also found that 81 proteins that were involved in response to various stresses tended to be differentially abundant in “*Yuza 1*” autotetraploids compared with its diploid parent. For instance, during these proteins three monocopper oxidase-like proteins (XP_002266352.1, XP_002329905.1, XP_004234931.1) and one L-ascorbate peroxidase (XP_002513962.1), which play important roles in the process of paulownia development and stress tolerance, turned out higher abundance levels in “*Yuza 1*” autotetraploid compared with its parents. These findings suggest that the regulation of protein abundance in autotetraploid “*Yuza 1*” may make for the increased adaptation observed in autopolyploids.

In plants, it has been reported that the when some stress-responsive genes are constitutively expressed, accompanied fitness costs are usually observed (Shen et al., 2015). Therefore, when defense is not required, the costs to plants could be reduced by induced resistance (Heil, 2002; Heil and Baldwin, 2002; Accamando and Cronin, 2012). In *Arabidopsis thaliana* autotetraploids, it was found that the higher expression levels of genes that responded to stress might be linked with slow growth of the plant compared with its parents (Ng et al., 2012). Shen et al. also reported that, in a Brassica hexaploid, the up-regulation of stress responsive genes might slow down the growth of the plant by cellular and metabolic processes (Shen et al., 2015). Interestingly, in this study, we found that the higher abundance of stress-responsive proteins was accompanied by a higher level of autotetraploid plant growth compared with the parent. How the autotetraploid Paulownia coordinates these biological processes is still unclear and further studies are required to clarify the underlying mechanisms.

### Correlation between protein abundance and transcript expression is limited

To interpret biological processes, such as gene expression, protein interactions, and the structures and functions of cellular systems, it is essential to investigate the correlation of protein abundance and mRNA transcript levels. However, the coefficient of correlation between them has been found to be different among different studies and different plant tissues. In yeast, good correlation has been reported between transcript levels and protein abundances (Lackner et al., 2012), while in most plant tissues, such as *A. thaliana* leaves and roots (Lan et al., 2012; Ng et al., 2012), *Brassica napus* (Marmagne et al., 2010), *Tragopogon mirus* (Koh et al., 2012), and *Triticum aestivum* (Song et al., 2007), only limited correlation has been found. In general, low correlation between protein abundance and mRNA expression levels is more often observed and is more widespread. In a study conducted by Lan et al. RNA sequencing and iTRAQ-based proteomics were both employed to generate genomic expression and proteomic abundance data of Arabidopsis roots, and discordant changes were reported. However, interestingly, they found that for highly up-regulated genes, the concordance between expression levels and protein abundance was generally high (Lan et al., 2012). In our study, we used RNA sequencing data and iTRAQ-based quantitative proteomics to compare the transcript levels and protein abundances in “*Yuza 1*” autotetraploid and diploid Paulownia leaves. Among the 2963 proteins detected by iTRAQ, 1808 genes that encoded these proteins were detected in the RNA sequencing data. Among them, 37 up-regulated genes corresponded to the more abundant proteins, and 22 down-regulated genes corresponded to the less abundant proteins in the Y4 vs. Y2 comparison. The expressions of a further 48 genes showed an opposite trend to the abundances of the corresponding proteins. We found that most of the genes and corresponding proteins that exhibited the same trend at the transcriptional and translational levels, respectively, were related to response to stimulus or oxidation-reduction. In an earlier study, we also found that up-regulated genes in Paulownia autotetraploids were significantly related with response to stimulus (Li et al., 2014). These data imply that changes in transcript levels may be one of the main determinants for changes in protein abundances.

Several factors may explain the reported lack of concordance between protein abundance and mRNA expression levels. First, it is widely assumed that posttranscriptional regulation and posttranslational modifications can control translational efficiency, and that these processes could lead to discordance between mRNA expression levels and protein abundance. For example, small RNAs, including microRNAs, which regulate the expression of their target genes, play important roles in many biological processes (Ha et al., 2009). Second, the limitations of the different experimental techniques in detecting the expression of mRNAs and abundance of proteins could contribute in a major way to this problem. Third, the use of different plant tissues might lead to different results because the transcriptional and translational processes are both temporal and spatial. Proteomic studies on whole tissues may draw a more comprehensive picture, but studies on specific organelles will likely show differences more exactly. In summary, despite the limited correlation, integrated analyses of the transcriptome and the proteome data are essential for understanding the effects of polyploidization.

## Author contributions

GF conceived the experiments. YD wrote the paper. MD performed the experiments. ZZ analyzed the data. YD contributed reagents/materials/analysis tools.

## Funding

This work was supported by the Fund of the Transformation Project of the National Agricultural Scientific and Technological Achievement of China (2012GB2D000271), the Central Financial Forestry Science Promotion Project (GTH [2012]01), the Fund of the Technology Innovation Team Project of Zhengzhou (121PCXTD515), the Joint Funds of the National Natural Science Foundation of China (NSFC) (Grant No. U1204309), the Fund of the Research CooperationProject of Henan Province (Grant No. 152107000097) and the Fund of Zhongyuan Scholarship Foundation of Henan Province (122101110700).

### Conflict of interest statement

The authors declare that the research was conducted in the absence of any commercial or financial relationships that could be construed as a potential conflict of interest.

## Supplementary material

Table S1 **KEGG pathway analysis of all identified proteins for “*Yuza 1***.”

Table S2 **Differentially abundant proteins identified in the comparison of Y4 vs. Y2 Up-regulated proteins were marked in red while down-regulated ones in green**.

Table S3 **Gene Ontology analysis of differentially abundant proteins in the comparison of Y4 vs. Y2 321 proteins (69.33% of total) were categorized into 41 function groups**.

Table S4 **KEGG analysis of the identified differentially abundant proteins in the comparison of Y4 vs. Y2**.

Table S5 **Proteins changed noteworthyly in expression levels with corresponding in transcript level in the comparison of Y4 vs. Y2. Same trend were marked in red while opposite ones in green**.

Table S6 **Proteins showed no changes in their translational levels while noteworthy changes in transcript level in the comparison of Y4 vs. Y2**.

Table S7 **proteins showed noteworthy changes in their translational levels had no change in transcript level in the comparison of Y4 vs. Y2**.

Figure S1 **Principal Component Analysis (PCA) of Y4 and Y2 samples**.

Figure S2 **Classification of the clusters of orthologous groups (COG) for the Itraq-based proteotome of “*Yuza 1***.”
