# Supplementary figures and images for: Quantitative Proteomic and Transcriptomic Study on Autotetraploid Paulownia and Its Diploid Parent Reveal Key Metabolic Processes Associated with Paulownia Autotetraploidization
Source: Front Plant Sci. 2016 Jun 24;7:892. doi: 10.3389/fpls.2016.00892 (PMC4919355; doi:10.3389/fpls.2016.00892)

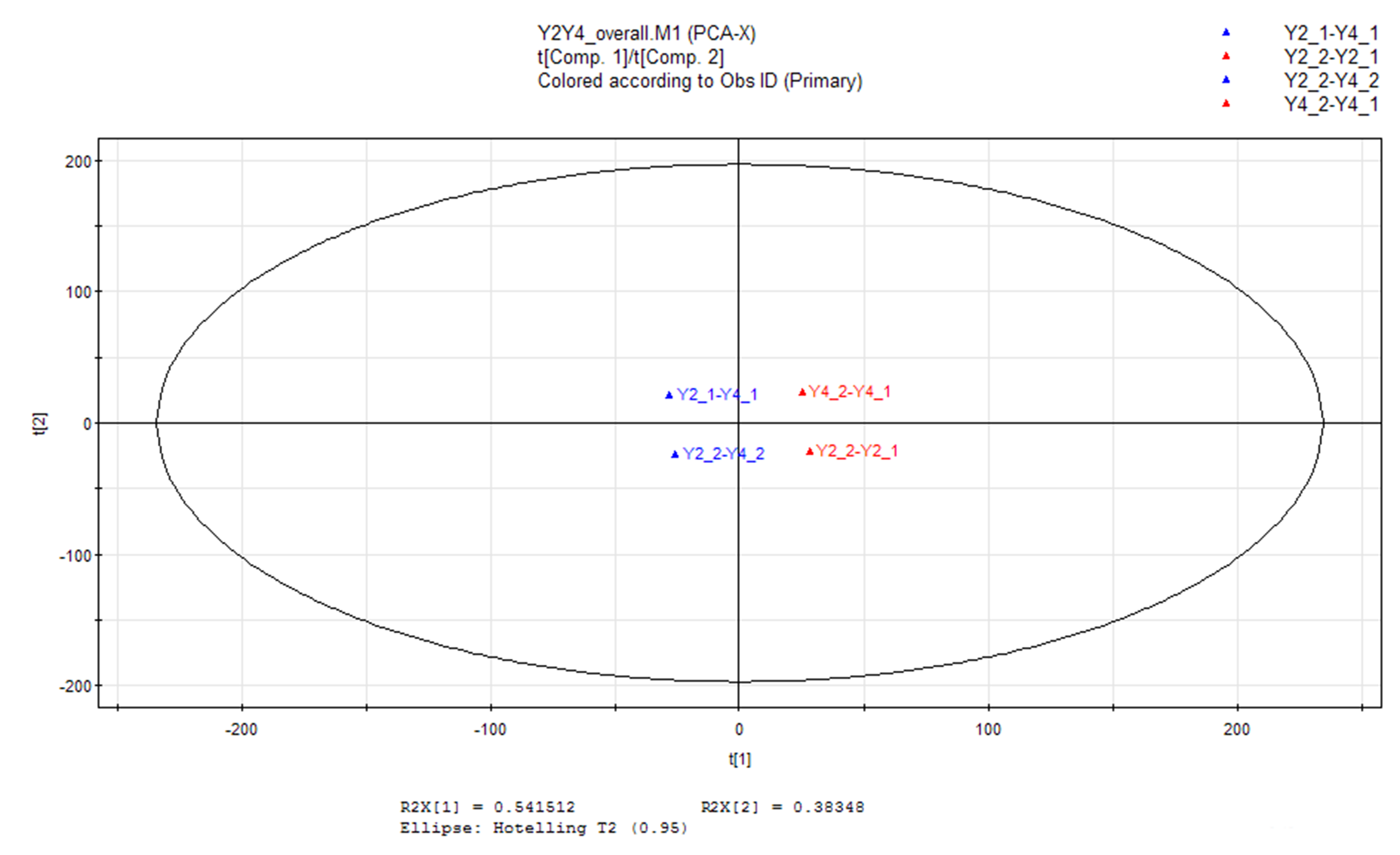

Supplement: Figure S1 — Principal Component Analysis (PCA) of Y4 and Y2 samples. [file Image1.TIF]

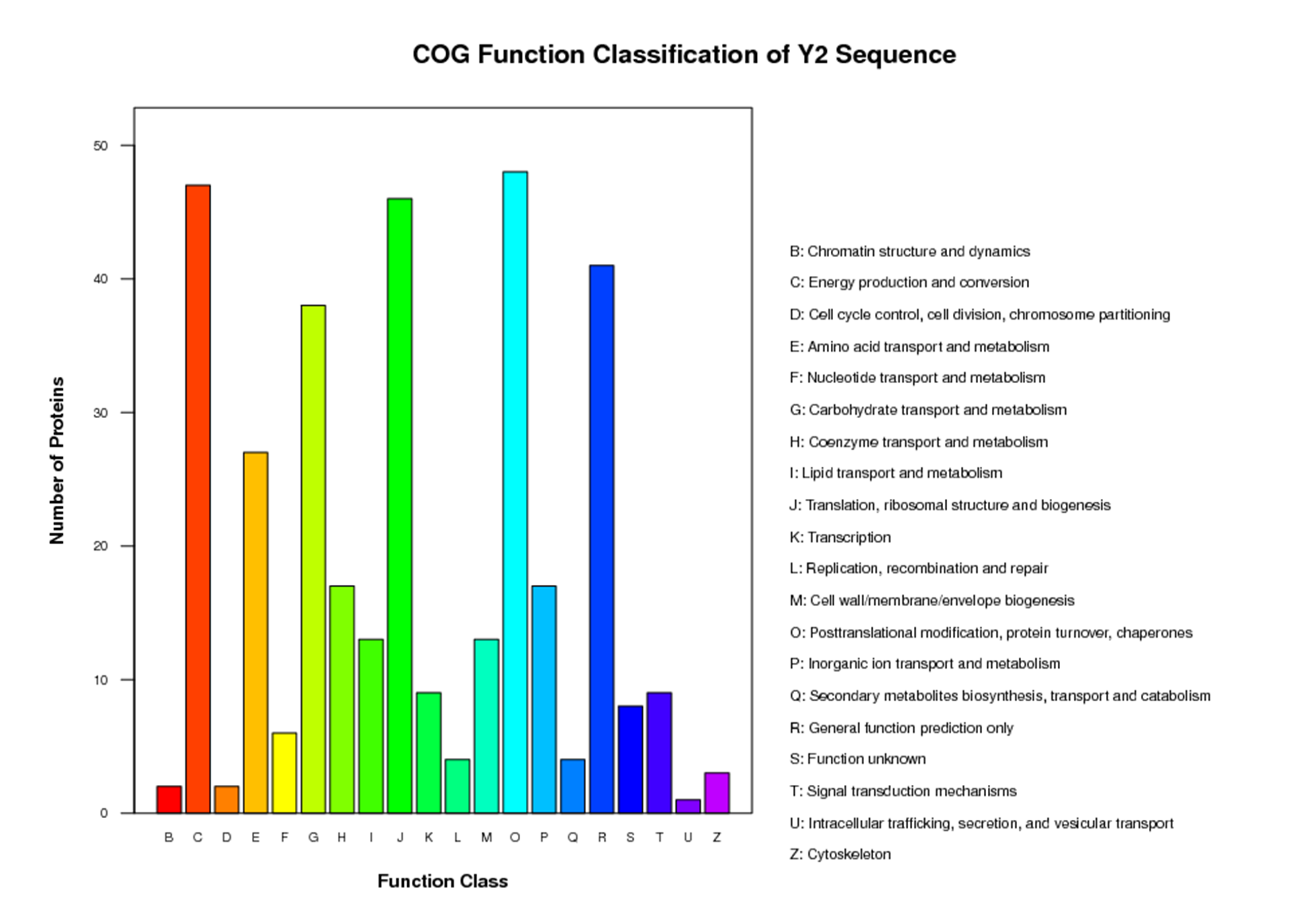

Supplement: Figure S2 — Classification of the clusters of orthologous groups (COG) for the Itraq-based proteotome of “Yuza 1.” [file Image2.TIF]
